# Microwave Ablation of Recurrent Hepatocellular Carcinoma after Curative Surgical Resection

**DOI:** 10.3390/jcm12072560

**Published:** 2023-03-29

**Authors:** Hamzah Adwan, Lars Hammann, Thomas J. Vogl

**Affiliations:** Department of Diagnostic and Interventional Radiology, University Hospital, Goethe University Frankfurt, Theodor-Stern-Kai 7, 60590 Frankfurt, Germany

**Keywords:** recurrent hepatocellular carcinoma, liver resection, microwave ablation

## Abstract

Purpose: To evaluate the efficacy and safety of microwave ablation (MWA) as a treatment for recurrent hepatocellular carcinoma (HCC) after initial successful surgical resection. Methods: This retrospective study included 40 patients (11 women and 29 men; mean age: 62.3 ± 11.7 years) with 48 recurrent lesions of HCC after initial surgical resection that were treated by percutaneous MWA. Several parameters including complications, technical success, local tumor progression (LTP), intrahepatic distant recurrence (IDR), overall survival (OS), and progression-free survival (PFS) were evaluated in order to investigate the safety and efficacy of MWA for these recurrent HCC lesions after surgical treatment. Results: All MWA treatments were performed without complications or procedure-related deaths. Technical success was achieved in all cases. Two cases developed LTP at a rate of 5%, and IDR occurred in 23 cases at a rate of 57.5% (23/40). The 1-, 2-, 3-, 4-, and 6-year OS rates were 97%, 89.2%, 80.3%, 70.2%, and 60.2%, respectively. The 1- and *3*-year PFS rates were 50.2% and 34.6%, respectively. Conclusion: MWA is effective and safe as a local treatment for recurrent HCC after initial surgical resection.

## 1. Introduction

Primary liver cancer, of which hepatocellular carcinoma (HCC) is the most common type, belongs to the most common newly diagnosed cancers and cancer-related deaths worldwide [1]. There are several available treatment options for HCC, including liver transplantation, surgical resection, local ablation, and transarterial chemoembolization (TACE) [2]. Liver transplantation and surgical resection are the treatments of choice for HCC in curative intent [3,4]. Despite the high efficacy of these surgical treatments, unfortunately, only less than 30% of patients with HCC are suitable for surgery [5]. One of the reasons that may limit the number of eligible patients for liver transplantations is that the MILAN criteria, being strict [6]. These criteria include a single HCC lesion with a diameter of ≤5 cm or up to 3 HCC lesions, each one measuring ≤ 3 cm [7]. The problem which faces HCC patients treated by liver resection is the development of recurrence, at high rates, of up to 50%–70% within five years [8,9]. Risk factors for recurrence after liver resection are various and consist of multinodular HCC [10], tumor size > 5 cm, as well as the presence of vascular invasion [10,11]. The high rates of recurrence raise the question which treatment options are most suitable for recurrent HCC after initial successful curative resection. It is principally possible to treat the recurrence of HCC with re-resection or with interventional treatments such as TACE or local ablation using RFA [12,13]. For primary HCC, local ablation, including radiofrequency ablation (RFA) or microwave ablation (MWA), is considered a suitable treatment for BCLC 0 and A stages [14]. The generated heat during MWA in the tumor tissue leads to necrosis and causes the tumor tissue to undergo demise [15,16].

As we know, there was only one study in the literature by Zhang et al. [17], which investigated the efficacy of percutaneous MWA and compared it with repeat hepatectomy for recurrent HCC after curative surgical resection on a long-term basis.

Therefore, this retrospective study focuses on evaluating the treatment of recurrent HCC after curative surgical resection by MWA, mainly according to complications, oncological outcome, and survival rates.

## 2. Materials and Methods

### 2.1. Ethics Committee Approval

This retrospective cohort study was approved by our institutional review board.

### 2.2. Patients

In this single-center study, a total of 40 patients (11 women and 29 men; mean age: 62.3 ± 11.7 years) with 48 recurrent HCC tumors were treated by percutaneous CT-guided MWA at our department for diagnostic and interventional radiology between 1 July 2013 and 1 April 2022, were enrolled. The inclusion criteria were (1) patients with recurrent HCC after curative R0 resection (2) patients with solitary recurrent tumor with a maximum axial diameter of <5 cm, (3) patients with a maximum number of 3 tumors each ≤3 cm, and (4) sufficient coagulation status. The exclusion criteria were (1) patients with R1 or R2 resection (2) patients who were treated by resection and ablation for the primary HCC before recurrence (3) patients with extrahepatic metastases, (4) patients with vascular invasion (5) decompensated liver function (Child Pugh class C) (6) patients with recurrent HCC, which were treated by TACE as a neoadjuvant treatment prior to MWA.

### 2.3. Protocol of MWA

Percutaneous MWA was performed under CT guidance (Siemens, Erlangen, Germany) by a radiologist with many years of experience in local ablation. All MWA sessions were carried out under conscious sedation and in concordance with device protocol settings. The latest abdominal contrast-enhanced cross-sectional imaging of the patients was evaluated, and recent hematological and coagulation parameters were verified prior to ablation. Each patient underwent an unenhanced abdominal CT scan before MWA to determine the best access way for the MWA antenna. Local anesthesia was carried out afterwards. When the local anesthesia had taken effect, the microwave antenna was inserted into the recurrent tumor under sterile conditions. After verification of the correct position of the antenna, subsequently, thermal ablation was carefully started. Fluoroscopic scans were used to continuously check the MWA process. After finishing the ablation session, the microwave antenna was pulled out, while sealing the needle track.

### 2.4. Follow-Up

For assessment of tumor response, contrast-enhanced MRI (Siemens, Erlangen, Germany) was performed by administration of intravenous contrast medium. We performed the first contrast-enhanced MRI 24 h post-ablation for evaluation of the ablation area. Patients underwent surveillance imaging every 3 months for the first year and every 6 months afterward to evaluate for tumor recurrence.

### 2.5. Parameters’ Analyzed and Definitions

Sex, age, etiology of liver disease, liver cirrhosis, Child Pugh class, surgical approach, time to recurrence after surgical resection, number and location of tumors, maximum axial diameters of tumor and ablation area, number of used antennas, ablation time, applied power, complications, technical success, LTP, IDR, follow-up time, overall survival (OS), and progression-free survival (PFS) were investigated in all patients’ cases. Complications were evaluated using the classification recommended by the Cardiovascular and Interventional Radiological Society of Europe (CIRSE) [18]. Time to recurrence after surgical resection was calculated from the date of surgery until the date of recurrence diagnosis. The ablation zone was measured using the largest axial diameter at the first MRI scan 24 h after MWA. Technical success was reached if MWA was carried out regarding to protocol and the recurrent tumor was completely covered by the ablation area 24 h after the MWA treatment at the first contrast-enhanced MRI scan [19]. LTP was defined as developing a new HCC lesion directly touching the ablation area in the follow-up [19]. IDR was defined as occurring of new HCC in other liver segments away from the initially ablated recurrent tumor. Hepatic complete response was achieved if the patient did not develop LTP or IDR after MWA. The OS was calculated from the date of MWA until the date of last contact or death. The PFS was calculated starting at the date of the MWA until the date of LTP or IDR or death. OS and PFS have also analyzed the presence of liver cirrhosis as well as the number of recurrent tumors.

### 2.6. Statistics

SPSS (Statistical Package for the Social Sciences) was used for the statistical analysis. Kaplan–Meier method was used to calculate and analyze the survival and the log–rank test was used for the comparison. A *p*-value of <0.05 was considered statistically significant. Quantitative variables are shown as average ± standard deviation. Categorical variables are presented using frequencies and percentages.

## 3. Results

The most common cause of liver disease was chronic viral hepatitis, followed by alcohol abuse at rates of 40% (16/40) and 22.5% (9/40), respectively. Liver cirrhosis was reported in 27 patients at a rate of 67.5%. Of these patients, 26 had a Child Pugh class A at a rate of 96.3%, and one had Child Pugh class B at a rate of 3.7%. Most surgical resections were performed by laparotomy at a rate of 77.5% (31/40). Laparoscopic surgical resection was carried out in 9 cases at a rate of 22%. The mean time to recurrence after surgical resection was 1.5 years. After curative surgical resection, a total of seven patients developed tumor recurrence within ≤6 months at a rate of 17.5%; 32.5% (13/40) of the patients had their tumor recurrence within 6 to ≤12 months. In 25% (10/40) of patients occurred, the recurrence within 12 to ≤24 months, and in another 25% (10/40) of the patients, after more than 24 months. Most of the included patients had solitary and small-sized (≤2 cm) recurrent tumors.

The rates of patients who had 1, 2, or 3 recurrent tumors were 82.5% (33/40), 15% (6/40), and 2.5% (1/40), respectively. The distribution of tumor size was as follows; 75% (36/48) of the tumors were smaller than 2 cm, 4.2% (2/48) were 2 cm, and 20.8% (10/48) were larger than 2 cm. The most recurrent tumors were located in the right liver lobe at the rate of 56.25% (27/48). The remaining 21 of 48 tumors were located in the left liver lobe at a rate of 43.75%. The average diameter of the recurrent tumor was 1.8 ± 0.8 cm. The patients’ and tumors’ characteristics are summarized in Table 1.

**Figure 1:**
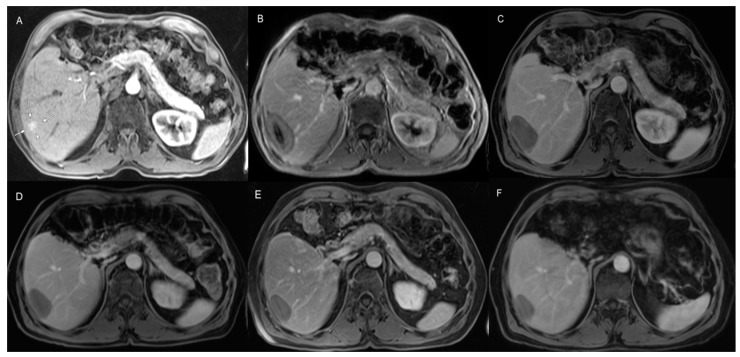
A 73-year-old male patient who developed intrahepatic recurrence 7 months after initially surgically resecting HCC. (**A**) pre-ablation contrast-enhanced MRI showed recurrent HCC lesion in liver segment 6 (white arrows). (**B**) A 24 h post-ablation MRI showed complete coverage of the recurrent tumor by the ablation area. (**C**) Three months post-ablation MRI. (**D**) Six months post-ablation MRI. (**E**) Twelve months post-ablation MRI. (**F**) Twenty-four months post-ablation MRI showed significant decrease in ablation area. This patient didn’t develop LTP or IDR during the follow-up period.

A total of 48 MWA treatments were performed. Technical success was achieved in all MWA treatments 100% (48/48). Each recurrent tumor was only treated by one MWA antenna. The average ablation time was 9 ± 2.5 min. The average applied power was 86 ± 20.7 watts. The average diameter of the ablation area was 4.5 ± 0.9 cm. There were no treatment-related complications or treatment-related deaths reported. A total of 15 patients at a rate of 37.5% showed complete hepatic response after MWA without developing IDR or LTP. Hepatic recurrence, including IDR and LTP, was observed in 25 patients at a rate of 62.5%. The rates of LTP and IDR were 5% (2/40), and 57.5% (23/40), respectively. The patients who developed recurrence after MWA were mainly treated by TACE and local ablation after thoroughly discussing the case at the multidisciplinary tumor board. Results are summarized in Table 2. Figure 1 shows a patient case.

**Figure 2:**
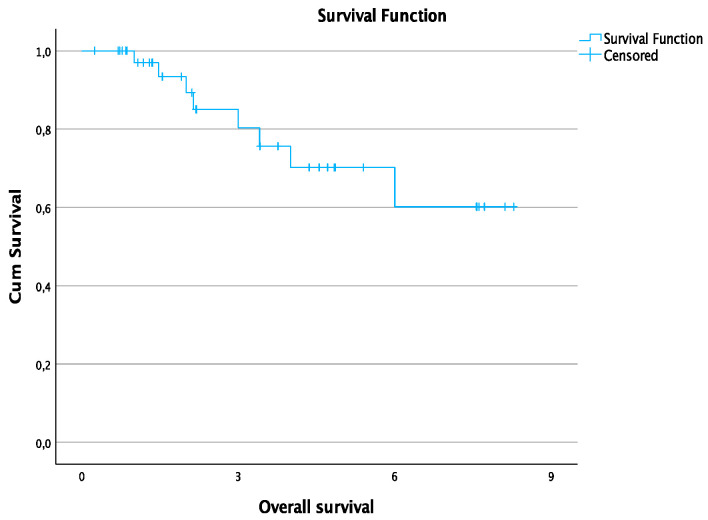
Overall survival for all patients in years.

**Figure 3:**
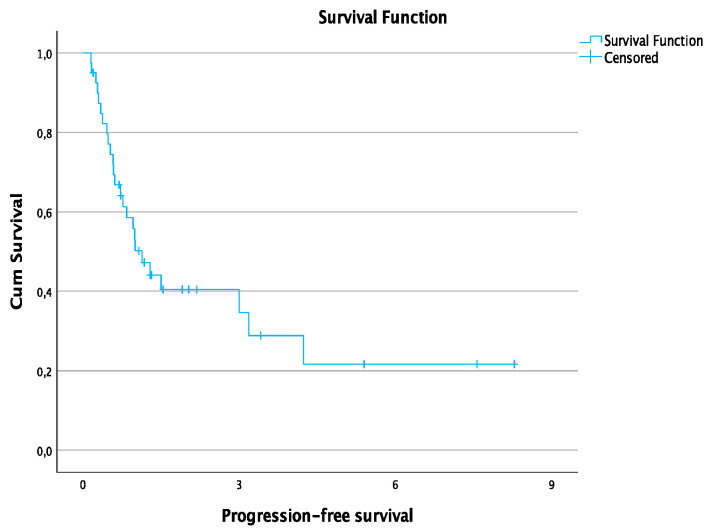
Progression-free survival for all patients in years.

The average follow-up time was 3.2 ± 2.5 years. The 1-, 2-, 3-, 4-, and 6-year OS rates were 97%, 89.2%, 80.3%, 70.2%, and 60.2%, respectively. The estimated median PFS time was 13.6 months. The 1-and 3-year PFS rates were 50.2% and 34.6%, respectively. Survival rates for all patients are summarized in Table 3. Figure 2 shows the curve of OS and Figure 3 shows the curve of PFS.

There was no statistically significant difference in OS (*p*-value: 0.1) or PFS (*p*-value: 0.5) between patients with or without liver cirrhosis. 

There was no statistically significant difference in OS (*p*-value: 0.6) or PFS (*p*-value: 0.7) between patients with solitary recurrent HCC and patients with multiple recurrent HCC lesions.

## 4. Discussion

Liver transplantation is an optimal curative treatment for HCC since it not only treats the tumor but also chronic liver disease [20,21]. Liver transplantation also provides high OS rates [22], with a low mean recurrence rate of 16% as shown by a systemic review that included 61 studies [23]. Tumor recurrence after liver transplantation occurs in most cases within the first 24 months after transplantation [24]. There are several factors that generally make liver transplantation very challenging, such as the limited number of donor organs [25,26] and the extended waiting time [27].

Unfortunately, there are no established guidelines or standards for the management of recurrent HCC. Surgical treatment can be conducted in only 20% of patients with HCC recurrence [28]. Patients who may be suitable for re-resection should have preserved hepatic function and enough remnant volume of the liver post repeat resection [28]. However, it is important to mention that in the case of recurrent HCC, patients have worse hepatic function and less future liver remnant in comparison to primary HCC [13]. Another main problem in treating a patient with re-resection is the higher possibility for hemorrhage during the repetitive surgery because of the adhesions caused by the primary surgery [29].

TACE, which has a wide range of indications for patients with HCC including neoadjuvant, bridging and palliative among others [30], is the most common used type of therapy for postoperative HCC recurrence after surgical resection [31]. 

When it comes to local thermal ablation, MWA has several advantages over RFA. Using MWA, higher temperatures can be achieved in tumor tissue, and it generates a larger ablation [32]. This study evaluated the efficacy and safety of thermal ablation as a local treatment for recurrent HCC after curative surgical resection, using percutaneous CT-guided MWA mainly according to complication, LTP, IDR, OS, and PFS. We showed that patients with recurrent HCC treated by MWA, had achieved long OS and median PFS time. The rate of developed LTP was low at 5%. IDR occurred in 57.5% of the patients. 

Lee et al. [33] analyzed, in their study, surgical resection and RFA for patients with Child Pugh class A and solitary small (≤3 cm) HCC as first-line treatments. They found that both RFA and surgical resection provides comparable OS. However, recurrence-free survival was better in patients treated by surgical resection. Eisele et al. [34] compared in their single-center study RFA with re-resection for recurrent HCC. They did not show a significant difference according to survival or disease-free survival between both treatment options. 

An advantage of local ablation over surgical therapy is the higher organ preservation and, thus, the better-preserved liver function posttheraputical [35]. Feng et al. [35] compared in their multicenter retrospective study also RFA with surgical resection as treatment for recurrent HCC after curative surgical resection. The 1- and 3-year OS rates were 90.7% and 69% in the RFA group and 87.7%, and 62.9% in the repetitive resection group, respectively. The corresponding PFS rates were 56.5%, and 27.9%, and 50.2%, and 21.9%, in the RFA group and repetitive resection group, respectively, without significant differences in the OS or PFS. The 1- and 3-year OS rates were 97% and 80.3%, and 1- and 3-year PFS rates were 50.2% and 34.6%, respectively, in our study. They also showed that RFA provided significantly longer OS compared to repeat resection in patients with two or three recurrent tumors. It was also better than repeat resection when it comes to complication rate and hospitalization. In our study, we did not report any complications or peri-procedural deaths. Chen et al. [36] compared TACE with RFA in treating recurrent HCC after resection in patients with stage BCLC 0/A. No significant difference in OS was observed between both treatments. However, RFA provided significantly higher OS in patients with BCLC stage 0. Recurrent HCC can also be treated using combination therapy. 

Peng et al. [37] showed that the combination therapy of TACE and RFA was superior to RFA alone in treating recurrent HCC. The 1-, 3-, and 5-year OS rates were 94%, 69%, and 46%, respectively, for the TACE/RFA group and 82%, 47%, and 36% for the RFA group. As mentioned, we only found one study which evaluated the percutaneous performance of MWA for recurrent HCC after surgical resection by Zhang et al. [17]. The 1- and 3-year OS rates were 86.2% and 73.3, respectively, for the MWA group and 96.2%, and 76.9%, respectively, for the re-resection group. Our OS rates at 1 and 3 years were higher. 

This study was limited by some points and factors. As a single-center retrospective study, the patient population was not large, and selection bias could occur. In addition, this study did not include a control group and did not directly compare MWA with any other possible and available treatments for recurrent HCC, such as RFA, TACE, or repetitive surgical resection, for instance. Another limitation was that this study did not focus on the prognostic factor for the outcome and survival of HCC patients with recurrent HCC that were treated by MWA. A comparative prospective randomized study with a larger number of patients and the presence of a control group should be conducted in the future for better analysis and investigation of different treatments for recurrent HCC after initial surgical resection.

## 5. Conclusions

In conclusion, we show that MWA is an effective, safe, and suitable treatment for recurrent HCC after curative surgical resection. It also represents a parenchyma-saving therapy for HCC, especially in previously surgically treated patients who already have reduced liver volume. MWA provided long OS and PFS without complications or long hospitalization stays and should be considered among the treatments for managing recurrent HCC after successful initial surgical resection.

## Figures and Tables

**Table 1:** Patients’ and tumors’ characteristics.

Parameter	Result
Number of patients	40
Mean age	62.3 ± 11.7 years
Gender *n* (%)	
Women	11 (22.5)
Men	29 (77.5)
Etiology of liver disease *n* (%)	
Chronic viral hepatitis	16 (40)
Alcoholic liver disease	9 (22.5)
Non-alcoholic fatty liver disease	4 (10)
Chronic viral hepatitis and alcoholic liver disease	3 (7.5)
Other/Cryptogenic	8 (20)
Cirrhosis *n* (%)	27 (67.5)
Child-Pugh class A	26 (96.3)
Child-Pugh class B	1 (3.7)
Surgical approach *n* (%)	
Laparotomy	31 (77.5)
Laparoscopic	9 (22.5)
Time to recurrence after surgical resection	1.5 years
Recurrence within ≤6 months *n* (%)	7 (17.5)
Recurrence within 6 to ≤12 months *n* (%)	13 (32.5)
Recurrence within 12 to ≤24 months *n* (%)	10 (25)
Recurrence after more than 24 months *n* (%)	10 (25)
No. of tumors	48
Tumor size	1.8 ± 0.8 cm
Distribution of recurrent tumors *n* (%)	
One tumor	33 (82.5)
Two tumors	6 (15)
Three tumors	1 (2.5)
<2 cm	36 (75)
=2 cm	2 (4.2)
>2 cm	10 (20.8)
Location *n* (%)	
Right lobe	27 (56.25)
Left lobe	21 (43.75)

**Table 2:** Results.

Parameter	Result
Diameter of ablation area	4.5 ± 0.9 cm
Number of microwave ablation treatments	48
Number of microwave ablation antennas	48
Technical success *n* (%)	48 (100)
Ablation time	9 ± 2.5 min
Power	86 ± 20.7 watts
Complete hepatic response *n* (%)	15 (37.5)
Local tumor progression *n* (%)	2 (5)
Intrahepatic distant recurrence *n* (%)	23 (57.5)
Treatment-related complications/deaths *n* (%)	0.0 (0.0)

**Table 3:** Survival for all patients.

Parameter	Result
Follow-up time	3.2 ± 2.5 years
1-year overall survival rate	97%
2-year overall survival rate	89.2%
3-year overall survival rate	80.3%
4-year overall survival rate	70.2%
6-year overall survival rate	60.2%
Median progression-free survival time	13.6 months
1-year progression-free survival rate	50.2%
3-year progression-free survival rate	34.6%

## Data Availability

Data are available on request from the corresponding author.
